# Supplementary material for: A New NT4 Peptide-Based Drug Delivery System for Cancer Treatment
Source: Molecules. 2020 Feb 28;25(5):1088. doi: 10.3390/molecules25051088 (PMC7179244; doi:10.3390/molecules25051088)
Supplement: Supplementary file 1 [file molecules-25-01088-s001.pdf]

Supplementary materials

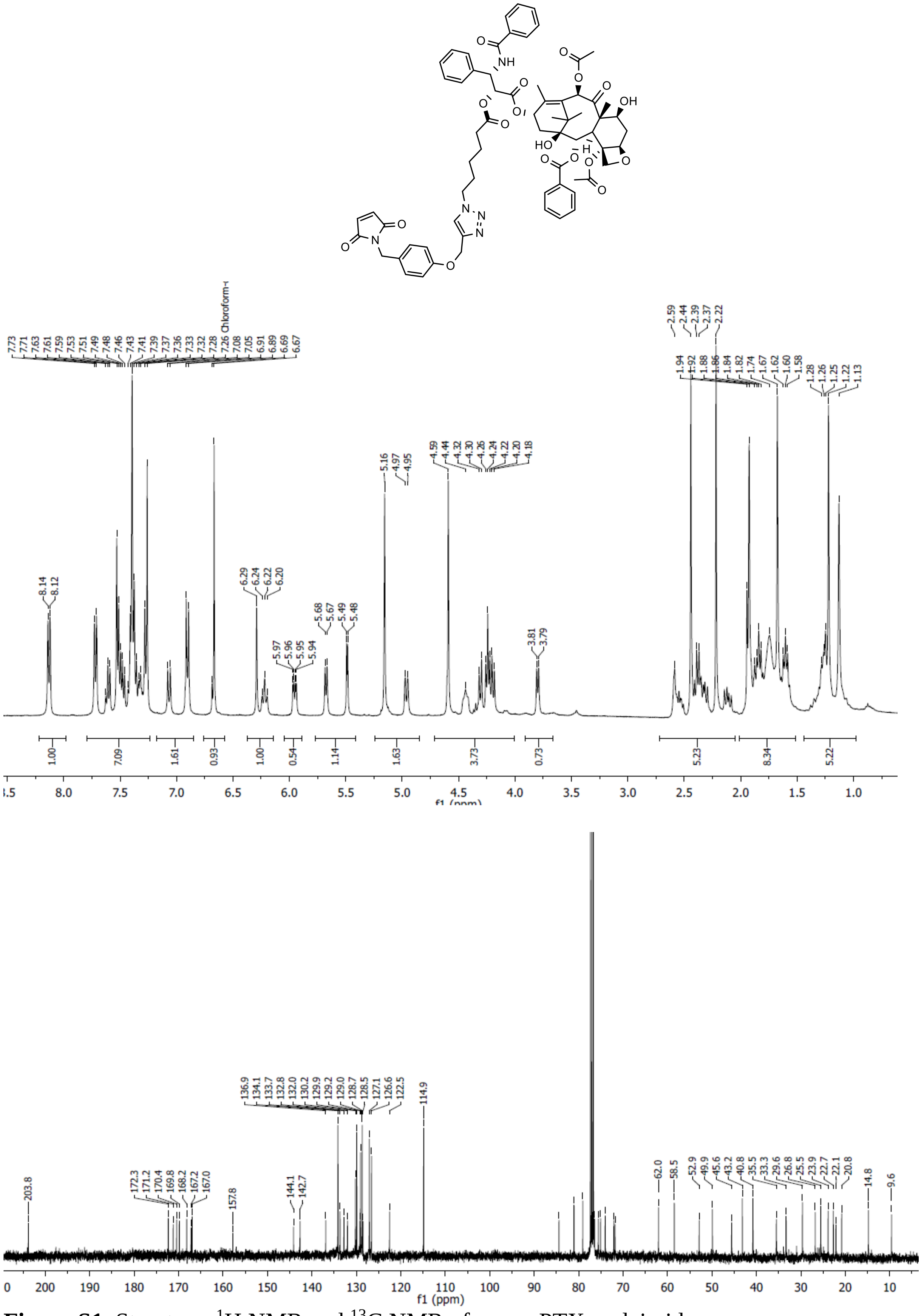

Figure S1. Structure, ^{1}H NMR and ^{13}C NMR of mono-PTX-maleimide

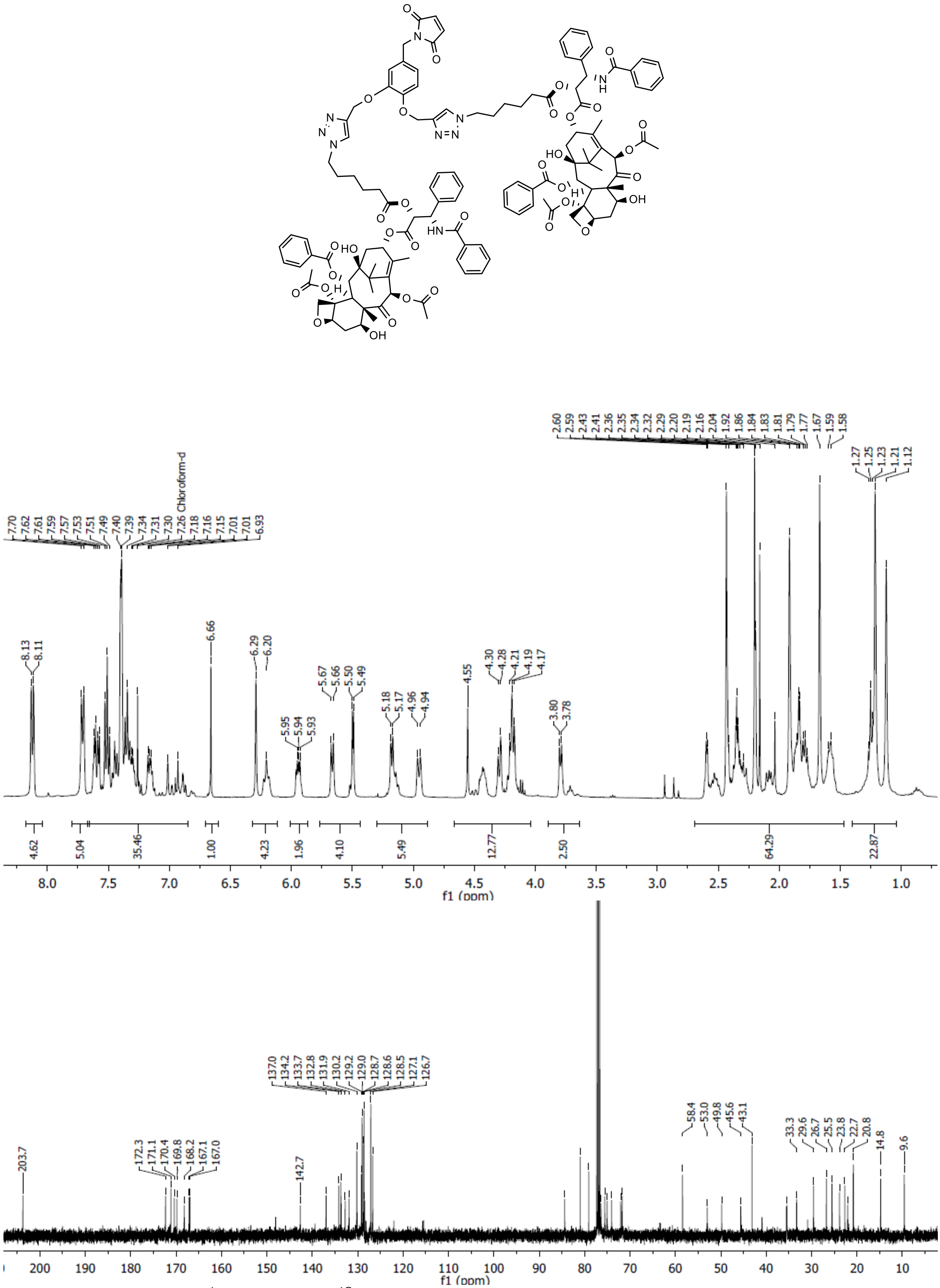

Figure S2. Structure, ^{1}H NMR and ^{13}C NMR of bis-PTX-maleimide

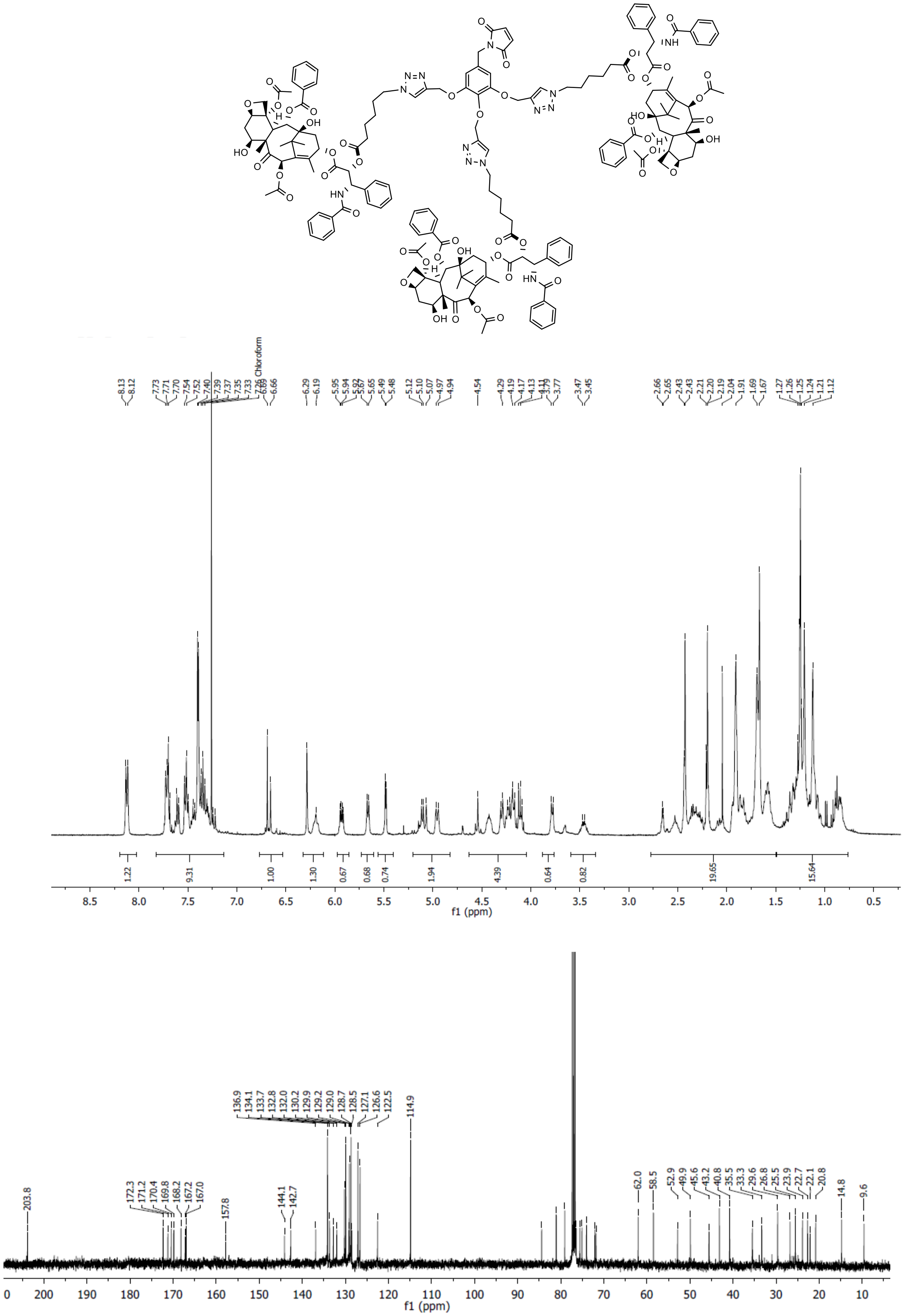

Figure S3. Structure, ^{1}H NMR and ^{13}C NMR of tris-PTX-maleimide

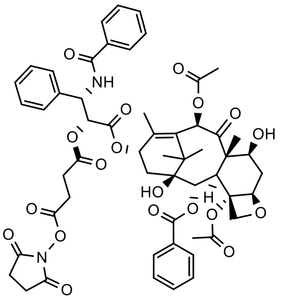

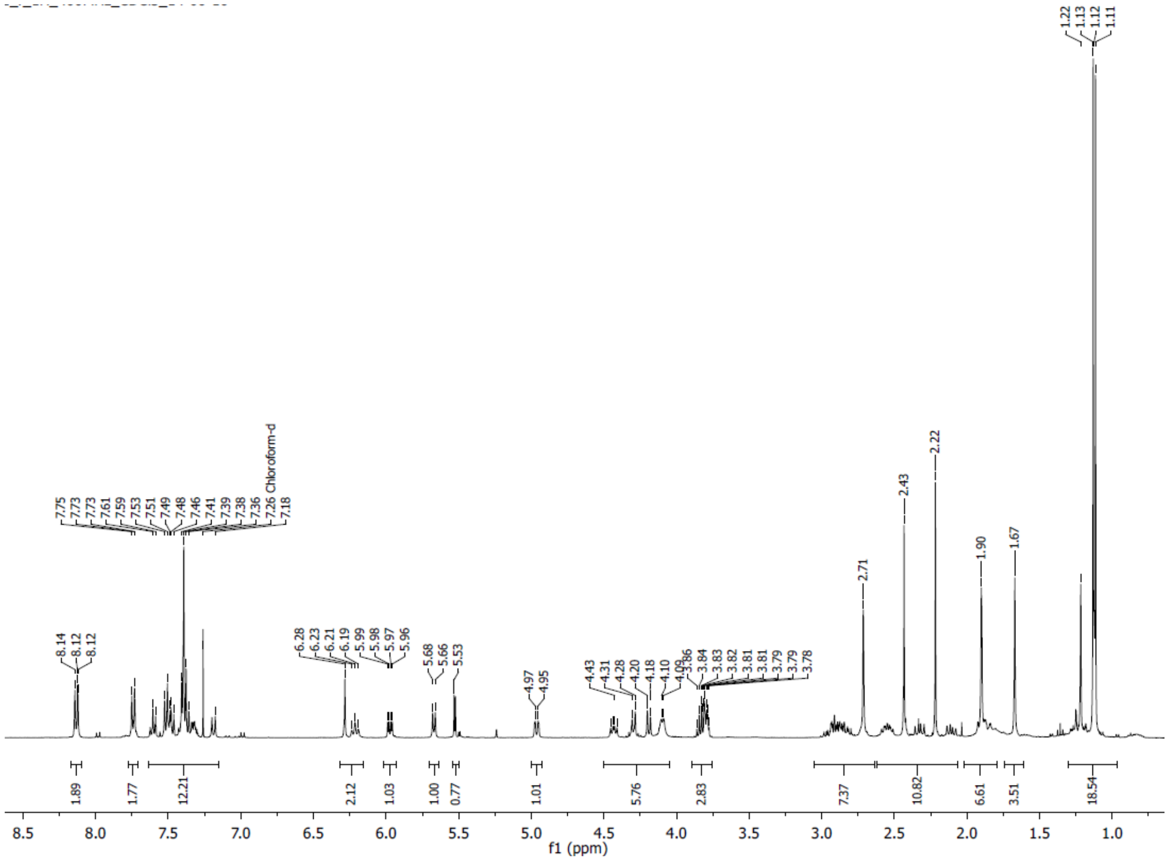

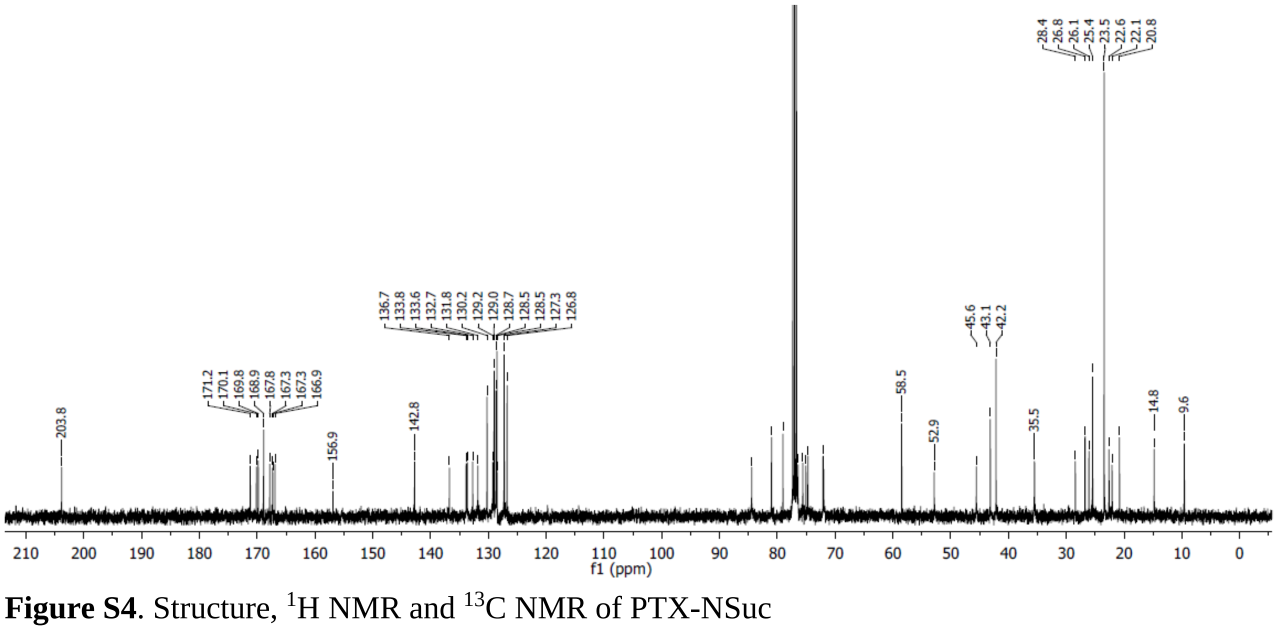

Figure S4. Structure, ^{1}H NMR and ^{13}C NMR of PTX-NSuc
